# The c-MYC-ABCB5 axis plays a pivotal role in 5-fluorouracil resistance in human colon cancer cells

**DOI:** 10.1111/jcmm.12531

**Published:** 2015-02-17

**Authors:** Naruji Kugimiya, Arata Nishimoto, Tohru Hosoyama, Koji Ueno, Tadahiko Enoki, Tao-Sheng Li, Kimikazu Hamano

**Affiliations:** a Department of Surgery and Clinical Science, Yamaguchi University Graduate School of MedicineYamaguchi, Japan; b Department of Stem Cell Biology, Atomic Bomb Disease Institute, Nagasaki UniversityNagasaki, Japan

**Keywords:** c-MYC, ABCB5, 5-fluorouracil resistance, colon cancer

## Abstract

c-MYC overexpression is frequently observed in various cancers including colon cancer and regulates many biological activities such as aberrant cell proliferation, apoptosis, genomic instability, immortalization and drug resistance. However, the mechanism by which c-MYC confers drug resistance remains to be fully elucidated. In this study, we found that the *c-MYC* expression level in primary colorectal cancer tissues correlated with the recurrence rate following 5-fluorouracil (5-FU)-based adjuvant chemotherapy. Supporting this finding, overexpression of exogenous c-MYC increased the survival rate following 5-FU treatment in human colon cancer cells, and knockdown of endogenous c-MYC decreased it. Furthermore, c-MYC knockdown decreased the expression level of *ABCB5*, which is involved in 5-FU resistance. Using a chromatin immunoprecipitation assay, we found that c-MYC bound to the *ABCB5* promoter region. c-MYC inhibitor (10058-F4) treatment inhibited c-MYC binding to the *ABCB5* promoter, leading to a decrease in ABCB5 expression level. ABCB5 knockdown decreased the survival rate following 5-FU treatment as expected, and the ABCB5 expression level was increased in 5-FU-resistant human colon cancer cells. Finally, using a human colon cancer xenograft murine model, we found that the combined 5-FU and 10058-F4 treatment significantly decreased tumorigenicity in nude mice compared with 5-FU or 10058-F4 treatment alone. 10058-F4 treatment decreased the ABCB5 expression level in the presence or absence of 5-FU. In contrast, 5-FU treatment alone increased the ABCB5 expression level. Taken together, these results suggest that c-MYC confers resistance to 5-FU through regulating ABCB5 expression in human colon cancer cells.

## Introduction

Colorectal cancer is the most common malignant solid tumour worldwide, and one of the leading causes of cancer-related deaths. 5-fluorouracil (5-FU) is one of the main chemotherapeutic agents used to kill colon cancer cells. In patients with advanced colon cancer, 5-FU treatment reduces tumour size and prolongs survival. However, the frequent emergence and survival of 5-FU-resistant colon cancer cells often causes recurrence following 5-FU-based adjuvant chemotherapy. Therefore, uncovering the mechanism underlying the acquisition of 5-FU resistance might facilitate the identification of a predictive marker of poor prognosis for 5-FU treatment, and provide a new potential therapeutic target for the treatment of colorectal cancer.

The *MYC* family genes encode transcription factors that regulate cell cycle, cell growth, differentiation, apoptosis, transformation, genomic instability and angiogenesis 1,2. In particular, overexpression of c-MYC has been found in various cancer cells 2 including colorectal cancer cells 3,4 and is often associated with poor prognosis 5. Furthermore, c-MYC has been found to be involved in drug resistance. Tumour cells resistant to cisplatin chemotherapy *in vivo* display elevated c-myc expression 6, and c-myc antisense oligonucleotides sensitize human colorectal cancer cells to chemotherapeutic drugs 7. Recent study has been shown that c-MYC overexpression decreased the expression level of the bridging integrator 1, leading to increased poly (ADP-ribose) polymerase 1 (PARP1) activity and resistance to cisplatin 8. However, the mechanism by which c-MYC regulates drug resistance remains to be fully elucidated.

ATP-binding cassette (ABC) transporters are a family of transporter proteins that contribute to drug resistance *via* ATP-dependent drug efflux pumps. Some ABC transporters confer chemoresistance by causing the efflux of anti-cancer drugs 9,10, and their expression levels correlate with the disease-free survival rate of colorectal cancer patients after adjuvant chemotherapy 11. Interestingly, recent studies have revealed that MYCN regulates the expression levels of some *ABC* transporter genes in neuroblastoma 12, and c-MYC regulates the expression levels of some *ABC* transporter genes in chronic myelogenous leukaemia 13.

In this study, we identified *ABCB5* as a novel c-MYC target gene and examined the role of the c-MYC-ABCB5 axis in 5-FU resistance in human colon cancer cells.

## Materials and methods

### Clinical colorectal cancer specimens

Patients with colorectal cancer who underwent surgical treatment at Yamaguchi University and affiliated hospitals between April 2012 and September 2012 were enrolled in this study. Detailed information about these patients is presented in Table1. Resected tumour specimens were immediately taken from resected colons and kept at −80°C until total RNA extraction. These samples were used in accordance with institutional guidelines and the Helsinki Declaration after obtaining informed consent from all patients.

**Table 1:** Relationship between recurrence and clinicopathological characteristics of colorectal patients treated with 5-FU-based adjuvant chemotherapy after curative surgery

	Non-Recurrence	Recurrence	
No. of patients	13	7	*P*
Gender			
Male	7 (53.8%)	3 (42.9%)	N.S.
Female	6 (46.2%)	4 (57.1%)	
Age	71.1 ± 8.1	75.0 ± 4.0	N.S.
Location			
Right	7 (53.8%)	2 (28.6%)	N.S.
Left	2 (15.4%)	2 (28.6%)	
Rectum	4 (30.8%)	3 (42.8%)	
Histological grade			
Well	1 (7.7%)	1 (14.3%)	N.S.
Moderate	12 (92.3%)	5 (71.4%)	
Poor	0 (0%)	1 (14.3%)	
Invasion depth			
T2	1 (7.7%)	0 (0%)	N.S.
T3	12 (92.3%)	6 (85.7%)	
T4	0 (0%)	1 (14.3%)	
Lymphatic metastasis			
Positive	7 (53.8%)	5 (71.4%)	N.S.
Negative	6 (46.2%)	2 (28.6%)	
Lymphatic invasion			
Positive	13 (100%)	5 (71.4%)	N.S.
Negative	0 (0%)	2 (28.6%)	
Venous invasion			
Positive	8 (61.5%)	3 (42.8%)	N.S.
Negative	5 (38.5%)	4 (57.1%)	
Stage (UICC, 2009)			
IIA	3 (23.1%)	0 (0%)	N.S.
IIB	3 (23.1%)	2 (28.6%)	
IIIA	1 (7.7%)	0 (0%)	
IIIB	2 (15.4%)	3 (42.8%)	
IIIC	4 (30.8%)	2 (28.6%)	

### Real-time quantitative reverse transcription- polymerase chain reaction (RT-PCR)

Resected tumour specimens were disrupted in Buffer RLT and homogenized with shaking stainless steel beads (Qiagen, Valencia, CA, USA) using Mixer Mill MM300 (Qiagen). After that, total RNA isolation was performed with RNeasy Mini Kit according to the manufacturer’s protocol (Qiagen). Reverse transcription was performed with the PrimeScript® RT Master Mix (Perfect Real Time; TaKaRa, Shiga, Japan). The cDNA template was amplified by real-time RT-PCR using the QuantiTect SYBR Green PCR kit (Qiagen). The primers used are listed in Table S1. The reaction condition was 95°C for 15 min., and followed by 50 cycles of the following reaction: 95°C for 10 sec. and 60°C for 30 sec. The quantitative RT-PCR was performed with LightCycler software ver 3.5 (Roche Applied Science, Tokyo, Japan) and data were evaluated using the 2^−ΔΔ*C*T^ method 14.

### Cell culture

The human colon cancer cell lines COLO-320, COLO205 and Caco-2 were purchased from the RIKEN Bioresource Center (Ibaraki, Japan). These cell lines were maintained at 37°C in RPMI 1640 medium (COLO-320 and COLO205) or MEM (Caco-2) containing 10% foetal calf serum (FCS) in a humidified atmosphere with 5% CO_2_. To establish 5-FU-resistant Caco-2 cells, Caco-2 cells were maintained in MEM containing 10% FCS with continuous exposure to 5-FU at the concentration of 2 μM for 12 weeks.

### Reagents

5-fluorouracil and cis-Diammineplatinum (II) dichloride (cisplatin) were purchased from Sigma-Aldrich (St. Louis, MO, USA). 10058-F4 was purchased from Calbiochem (San Diego, CA, USA). 5-FU and 10058-F4 were dissolved in dimethyl sulfoxide (DMSO) and stored at 4°C. Cisplatin was dissolved in 0.9% sodium chloride just before use and stored at 4°C.

### Knockdown of target gene expression by small interfering RNA transfection

Scrambled (control), *c-MYC* small interfering RNA (siRNA; Thermo Scientific Dharmacon, Lafayette, CO, USA), *ABCB5* siRNA (Life Technologies, Carlsbad, CA, USA) or *ABCC5* siRNA (Life Technologies) was transfected as described previously 15.

### Overexpression of exogenous c-MYC

We purchased a pcDNA3 vector containing a full-length cDNA that encodes human *c-MYC* (pcDNA3-*c-MYC*) from Addgene (Cambridge, MA, USA), and the pcDNA3 empty vector was kindly provided by the Yamaguchi University Center for Gene Research. Transfection of pcDNA3-*c-MYC* or pcDNA3 empty vector into COLO205 cells was performed with Lipofectamine 2000 (Life Technologies) according to the manufacturer’s protocol.

### Preparation of nuclear extracts and whole cell lysates

Nuclear extracts were prepared from COLO205 and COLO-320 cells using Nuclear Complex Co-IP Kit (Active Motif, Carlsbad, CA, USA) according to the manufacturer’s protocol. Whole cell lysates from COLO-320, Caco-2 and 5-FU-resistant Caco-2 cells were prepared using 1% (v/v) Nonidet P-40 (NP-40) lysis buffer as described previously 16. Caco-2 xenografts was lyzed with RIPA buffer (50 mM Tris-HCl (pH 7.4), 150 mM NaCl, 1% (v/v) NP-40, 0.5% sodium deoxycholate, 0.1% SDS, 1 X protease inhibitor cocktail) and homogenized with shaking stainless steel beads (Qiagen) using Mixer Mill MM300 (Qiagen).

### Western blotting

Western blotting was performed as described previously 16 using nuclear extracts or whole cell lysates. The following primary and secondary antibodies were used. The primary antibodies were mouse monoclonal c-MYC antibody (9E10; Santa Cruz Biotechnology, Inc., Dallas, TX, USA), goat polyclonal ABCB5 antibody (46-620; ProSci Inc., Poway, CA, USA), goat polyclonal MRP5 antibody (Abcam, Cambridge, MA, USA), rabbit monoclonal BCRP/ABCG2 antibody (Abcam), rabbit monoclonal PCNA antibody (D3H8P XP; Cell Signaling, Danvers, MA, USA), mouse monoclonal PARP1 antibody (F-2; Santa Cruz Biotechnology, Inc.) and mouse monoclonal α-Tubulin antibody (B-7; Santa Cruz Biotechnology, Inc.). The secondary antibodies were antimouse immunoglobulins conjugated to horseradish peroxidase (Igs-HRP), anti-rabbit Igs-HRP and anti-goat Igs-HRP (Dako, Carpinteria, CA, USA).

### Cell viability assay (WST-8 assay)

Cell viability was evaluated using Cell Count Reagent SF (Nacalai Tesque; Kyoto, Japan) according to the manufacturer’s protocol. Cell viability was determined by measuring the absorbance at 450 nm using a microplate reader (Model 550; Bio-Rad Laboratories, Hercules, CA, USA).

### Semi-quantitative RT-PCR

Total RNA isolation, synthesis of cDNA and subsequent PCR were performed as described previously 15. DNA sequences of the primers used for RT-PCR are listed in Table S2. Reaction conditions for each primer set were 50°C for 30 min. and 94°C for 2 min. followed by 20 cycles (*MDR1*, *GAPDH*), 24 cycles (*c-MYC*, *ABCB5*), 25 cycles (*ABCC4*, *ABCC5*) or 27 cycles (*MRP1*) of the following reaction: denaturing step at 94°C for 30 sec.; annealing at 54.5°C (*ABCB5*), 55°C (*GAPDH*), 57.5°C (*c-MYC*) or 60°C (*MDR1*, *MRP1*, *ABCC4*, *ABCC5*) for 30 sec.; and extension at 72°C for 30 sec. PCR products were analyzed on a 1% agarose gel containing ethidium bromide and detected using a UV transilluminator.

### Chromatin immunoprecipitation

Chromatin immunoprecipitation (ChIP) was performed with ChIP-IT™ Express Enzymatic (Active Motif) according to the manufacturer’s protocol. Normal mouse IgG (sc-2027) and mouse monoclonal anti-c-MYC (N-262) antibody were used for ChIP (Santa Cruz Biotechnology, Inc.). Each region containing a c-MYC-binding site within the *ABCB5* promoter was amplified using each primer set. DNA sequences of the primers used for the ChIP assay are listed in Table S3. The reaction condition was 28 cycles of denaturation at 94°C for 20 sec., annealing at 54°C for 30 sec. and extension at 72°C for 30 sec. PCR products were resolved on a 3% agarose gel containing ethidium bromide and detected using an ultraviolet transilluminator.

### Nude mouse xenograft model and *in vivo* experiments

Seven-week-old female BALB/c athymic Nu/Nu mice were purchased from Japan SLC Inc. (Shizuoka, Japan). A mixture of 1 × 10^6^ Caco-2 cells and Matrigel (BD Biosciences, San Jose, CA, USA) was subcutaneously injected to the right side of the back of the mice under anaesthesia. Tumour size was measured using a caliper, and the volume was calculated using the following formula: volume = length × width × height. When the tumour size was about 1000 mm^3^, the mice were daily treated with DMSO as a vehicle control, 5-FU (10 mg/kg), 10058-F4 (20 mg/kg) or 5-FU (10 mg/kg) and 10058-F4 (20 mg/kg) by intraperitoneal administration for 2 weeks. The tumour volume was measured on day 0, 3, 5, 7, 10 and 14 following each treatment. Tumour tissues were used for immunofluorescent and western blotting analyses. All animal experiments in this study were approved by the Institutional Animal Care and Use Committee of Yamaguchi University.

### Immunofluorescence staining

The excised human colon cancer xenografts were fixed with a 10% formalin neutral buffer solution (Wako; Osaka, Japan) for 24 hrs at room temperature and were placed in 10% sucrose, 20% sucrose and 30% sucrose for 6 hrs each at 4°C with shaking. The samples were embedded in OCT compound, and the embedded samples were sectioned (5 μm) for immunofluorescence staining. The sections were first incubated with blocking reagent including 1% Triton X-100 for 1 hr at room temperature. After aspirating the blocking solution, the sections were incubated with a rabbit monoclonal antibody against Ki67 [SP6] (Abcam) or a goat polyclonal antibody against ABCB5 (Abcam) at 4°C overnight. The sections were rinsed three times with 1× PBS for 5 min. each and were incubated with Alexa Fluor 555F (ab’)_2_ fragment of goat anti-rabbit IgG (H+L) (Life Technologies) or donkey anti-goat IgG-TR (Santa Cruz Biotechnology, Inc.) for 1 hr at room temperature in the dark. The sections were rinsed three times with 1× PBS for 5 min. each. Nuclei were detected using DAPI staining. After washing three times with 1× PBS for 5 min. each, the sections were mounted with Fluorescent Mounting Medium (Dako). The images were acquired using the 360 ± 20 nm (blue) and 545 ± 12.5 nm (red) excitation filters on a BZ-X710 All-in-One fluorescence microscope (KEYENCE Japan, Osaka, Japan).

### Detection of apoptotic cells by TUNEL method

The paraffin tissue sections were deparaffinized by heating at room temperature for 5 min. to melt the wax, then immersed in xylene and washed in a graded ethanol. The sections were rinsed three times with 1× PBS for 5 min. each and were incubated with proteinase K at 37°C for 30 min., and then with PBS including 0.1% Triton X-100 for 2 min. After washing with PBS, apoptotic cells were detected with TUNEL method using *In situ* Cell Death Detection Kit, TMR red (Roche Applied Science, Waltham, MA, USA) according to the manufacturer’s protocol. Nuclei were detected using DAPI staining. After washing three times with 1× PBS for 5 min. each, the sections were mounted with Fluorescent Mounting Medium (Dako). The images were acquired using the 360 ± 20 nm (blue) and 545 ± 12.5 nm (red) excitation filters on a BZ-X710 All-in-One fluorescence microscope (KEYENCE Japan).

### Detection of apoptotic dead cells by flow cytometric analysis

Caco-2 cells were treated with DMSO, 5-FU (10 μM), 10058-F4 (32 μM), 5-FU (10 μM) and 10058-F4 (32 μM) for 72 hrs. The cells were resuspended in binding buffer containing Annexin V-FITC and PI using FITC Annexin V Apoptosis Detection Kit I (BD Biosciences) according to the manufacturer’s protocol. Annexin V-FITC and PI staining were quantified using a FACSCalibur flow cytometer (Becton Dickenson, Franklin Lakes, NJ, USA).

### Statistical analysis

Statistical analyses were performed with SPSS for Windows ver. II. Data are expressed as means ± SD. To compare the experimental groups, Student’s *t*-test was performed. Data from clinical colorectal cancer specimens were analysed using Pearson correlation calculations. Kaplan–Meier curves were compared using the log-rank test. *P* < 0.05 (∗) or *P* < 0.01 (∗∗) was considered to be statistically significant.

## Results

### High expression of *c-MYC* in surgically resected primary colorectal cancer tissues correlates with recurrence following 5-FU-based adjuvant chemotherapy

**Figure 1:**
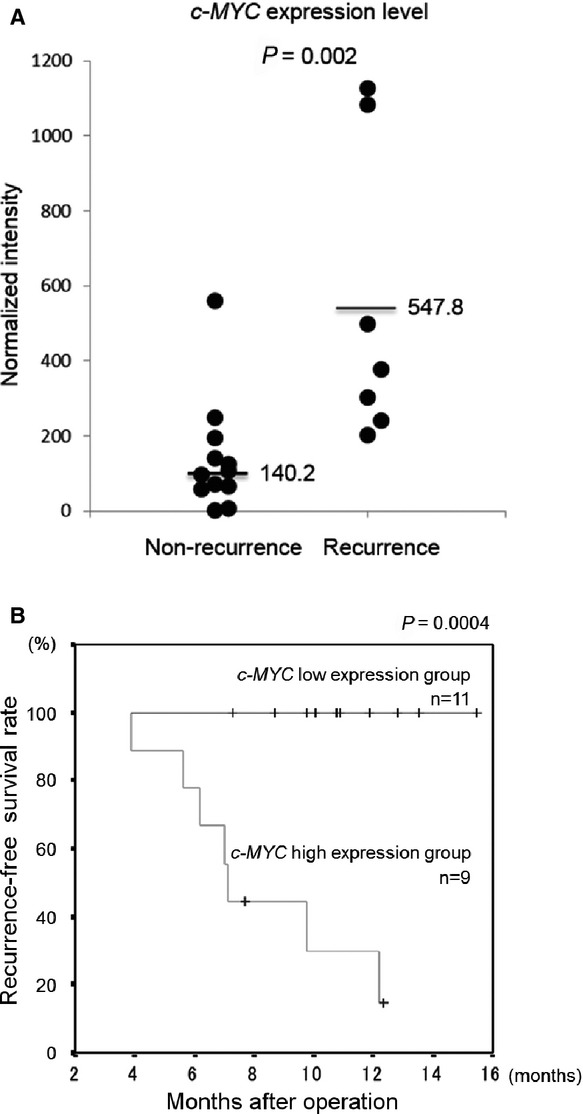
Relationship between *c-MYC* expression level in surgically resected primary colorectal cancer tissues and recurrence following 5-FU-based adjuvant chemotherapy. (A) *c-MYC* expression levels in clinical colorectal cancer tissues were determined by real-time RT-PCR, and were compared between the recurrence (*n* = 7) and non-recurrence group (*n* = 13) following 5-FU-based adjuvant chemotherapy. The ratio of *c-MYC* to *GAPDH* expression was normalized to the lowest value obtained in non-recurrence group. Horizontal lines indicate the mean *c-MYC* expression level. (B) Kaplan–Meier analysis for recurrence-free survival rate following 5-FU-based adjuvant chemotherapy.

To investigate whether *c-MYC* expression in surgically resected primary colorectal cancer tissues correlates with recurrence following 5-FU-based adjuvant chemotherapy, we selected 20 patients who had received 5-FU-based adjuvant chemotherapy after curative surgery. Total RNAs were purified from surgically resected tumours before 5-FU-based adjuvant chemotherapy and used for real-time RT-PCR. As shown in Figure1A, the *c-MYC* expression level in surgically resected primary colorectal cancer tissues of patients with recurrence following 5-FU-based adjuvant chemotherapy was significantly higher than that of patients without recurrence (*P* = 0.002). Clinicopathological characteristics of these colorectal cancer patients are presented in Table1. As shown in Table1, all clinicopathological characteristics did not correlate with recurrence following 5-FU-based adjuvant chemotherapy. Furthermore, ROC curve analysis was used to obtain the optimal cut-off value of *c-MYC* mRNA levels in primary surgical cancer tissues of colorectal cancer patients. As shown in Figure1B and Table2, the Kaplan–Meier plot showed that patients with the high expression level of *c-MYC* mRNA had a poorer recurrence-free survival rate than that of the patients with low expression level of *c-MYC* mRNA (*P* = 0.0004). These results suggest that the high *c-MYC* expression level in primary colorectal cancer tissues correlates with the recurrence rate following 5-FU-based adjuvant chemotherapy.

**Table 2:** Relationship between *c-MYC* expression and clinicopathological characteristics of colorectal patients treated with 5-FU-based adjuvant chemotherapy after curative surgery

*c-MYC* expression	Low	High	
No. of patients	11	9	*P*
Gender			
Male	7 (63.6%)	3 (33.3%)	N.S.
Female	4 (36.4%)	6 (66.7%)	
Age	70.8 ± 8.7	74.3 ± 3.7	N.S.
Location			
Right	6 (54.5%)	3 (33.3%)	N.S.
Left	1 (9.1%)	3 (33.3%)	
Rectum	4 (36.4%)	3 (33.3%)	
Histological grade			
Well	1 (9.1%)	1 (11.1%)	N.S.
Moderate	10 (90.9%)	7 (77.8%)	
Poor	0 (0%)	1 (11.1%)	
Invasion depth			
T2	0 (0%)	1 (11.1%)	N.S.
T3	11 (100%)	7 (77.8%)	
T4	0 (0%)	1 (11.1%)	
Lymphatic metastasis			
Positive	6 (54.5%)	6 (66.7%)	N.S.
Negative	5 (45.5%)	3 (33.3%)	
Lymphatic invasion			
Positive	11 (100%)	7 (77.8%)	N.S.
Negative	0 (0%)	2 (22.2%)	
Venous invasion			
Positive	7 (63.6%)	4 (44.4%)	N.S.
Negative	4 (36.4%)	5 (55.6%)	
Stage (UICC, 2009)			
IIA	2 (18.2%)	1 (11.1%)	N.S.
IIB	3 (27.3%)	2 (22.2%)	
IIIA	1 (9.1%)	0 (0%)	
IIIB	2 (18.2%)	3 (33.3%)	
IIIC	3 (27.3%)	3 (33.3%)	
Chemotherapy regimen			
UFT	4 (36.4%)	4 (44.4%)	N.S.
Xeloda	2 (18.2%)	2 (22.2%)	
TS-1	5 (45.5%)	3 (33.3%)	
Recurrence			
+	0 (0%)	7 (77.8%)	0.0002
−	11 (100%)	2 (22.2%)	

### c-MYC modulates 5-FU resistance in human colon cancer cells

**Figure 2:**
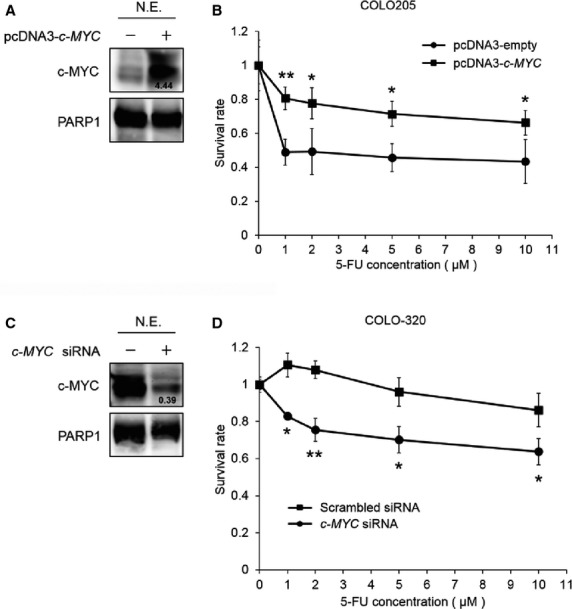
Effect of c-MYC overexpression or knockdown on the survival rate following 5-FU treatment. (A) Nuclear extracts were prepared from COLO205 cells transfected with pcDNA3 empty vector or pcDNA3-*c-MYC*, and nuclear c-MYC and PARP1 expression was detected by western blotting. PARP1 was used to assess the total amount of nuclear extracts loaded on the gel. The intensity of each band was quantified using ImageJ analysis software. The intensity of other bands was quantified in the same way. The number under the band in the upper right lane shows the ratio of c-MYC to PARP1 band intensity normalized to the value obtained in control cells. N.E., nuclear extracts. (B) COLO205 cells transfected with pcDNA3 empty vector or pcDNA3-*c-MYC* were treated with DMSO or 5-FU at various concentrations (1, 2, 5 and 10 μM) for 48 hrs, followed by a WST-8 assay to assess survival rates. The survival rate was normalized to that in the pcDNA3 empty vector or pcDNA3-*c-MYC* transfected cells treated with DMSO as a vehicle control. *P < 0.05; **P < 0.01, significantly different (*n* = 4). (C) Nuclear extracts were prepared from COLO-320 cells transfected with scrambled siRNA or *c-MYC* siRNA. Nuclear c-MYC and PARP1 expression was detected by western blotting. PARP1 was used to assess the total amount of nuclear extracts loaded on the gel. The number under the band in the upper right lane shows the ratio of c-MYC to PARP1 band intensity normalized to the value obtained in control cells. N.E., nuclear extracts. (D) COLO-320 cells transfected with scrambled siRNA or *c-MYC* siRNA were treated with DMSO or 5-FU at various concentrations (1, 2, 5 and 10 μM) for 48 hrs, followed by a WST-8 assay to assess the survival rate. The survival rate was normalized to that in the scrambled siRNA or *c-MYC* siRNA transfected cells treated with DMSO as a vehicle control. *P < 0.05; **P < 0.01, significantly different (*n* = 4).

To determine whether c-MYC is involved in 5-FU resistance, we investigated the effect of c-MYC overexpression or knockdown on the survival rate following 5-FU treatment in human colon cancer cells. We selected COLO205 cells for exogenous c-MYC overexpression, because parental COLO205 cells had low c-MYC expression level in the nucleus, but not in the cytoplasm (data not shown). COLO205 cells were transfected with pcDNA3-empty vector or pcDNA3-*c-MYC*, and exogenous c-MYC overexpression was confirmed by western blotting (Fig.2A). As shown in Figure2B, c-MYC overexpression in COLO205 cells significantly increased the survival rate following 5-FU treatment compared with control cells. Furthermore, we selected COLO-320 cells for knockdown of endogenous c-MYC, because parental COLO-320 cells presented a high c-MYC expression level in the nucleus, but not in the cytoplasm (data not shown). COLO-320 cells were transfected with scrambled siRNA or *c-MYC* siRNA, and the knockdown of endogenous c-MYC in nuclear extracts of COLO-320 cells was confirmed by western blotting (Fig.2C). As shown in Figure2D, c-MYC knockdown significantly decreased the survival rate following 5-FU treatment when compared with control cells. As expected, the survival rate following 5-FU treatment in parental COLO-320 cells was higher than that in parental COLO205 cells, and there was correlation between *c-MYC* expression level and the survival rate following 5-FU treatment (data not shown). These results indicate that c-MYC modulates 5-FU resistance in human colon cancer cells.

### c-MYC regulates *ABCB5* expression through its binding to the *ABCB5* promoter

**Figure 3:**
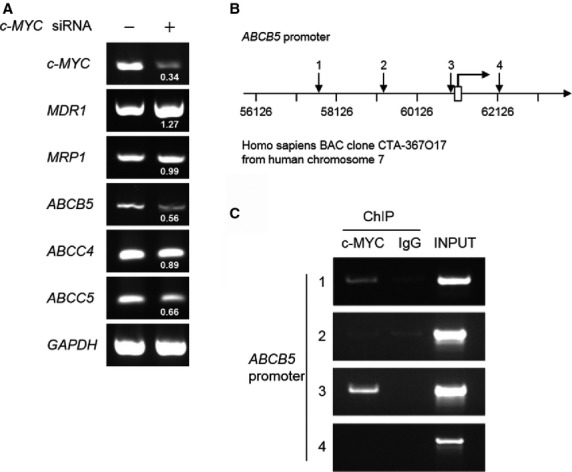
Regulation of *ABCB5* expression through c-MYC binding to the *ABCB5* promoter region. (A) Total RNAs were purified from COLO-320 cells transfected with scrambled siRNA or *c-MYC* siRNA, and *c-MYC*, *MDR1*, *MRP1*, *ABCB5*, *ABCC4*, *ABCC5* and *GAPDH* expression levels were detected by RT-PCR. The number under each band in the right lane shows the ratio of *c-MYC*, *MDR1*, *MRP1*, *ABCB5*, *ABCC4* or *ABCC5* to *GAPDH* band intensity normalized to the values obtained in control cells. (B) Schema of the putative c-MYC-binding sites within the *ABCB5* promoter region. The numbered black arrow, putative c-MYC binding site; bent arrow and open box, transcription start site. *Homo sapiens* BAC clone from human chromosome 7 and coordinates (bp) are also presented. (C) A ChIP assay was performed on COLO-320 cells in which endogenous c-MYC protein is expressed. The numbers on the left correspond to the arrow numbers in B. c-MYC, mouse monoclonal anti-c-MYC antibody; IgG, normal mouse IgG; INPUT, chromatin DNA of 1/100 quantity used in ChIP.

To identify the *ABC* transporter gene regulated directly by c-MYC, we investigated the effect of c-MYC knockdown on the expression levels of 5 *ABC* transporter genes, which are closely involved in resistance to chemotherapeutic agents including 5-FU 17–23. Total RNAs were purified from COLO-320 cells transfected with scrambled siRNA or *c-MYC* siRNA and used for RT-PCR to assess *MDR1*, *MRP1*, *ABCB5*, *ABCC4* and *ABCC5* expression levels. The results showed that c-MYC knockdown decreased *ABCB5*, *ABCC4* and *ABCC5* expression levels, whereas *MDR1* and *MRP1* expression levels increased or nearly remained unchanged (Fig.3A). Among *ABCB5*, *ABCC4*, and *ABCC5*, *ABCB5* expression level was decreased the most by c-MYC knockdown. Therefore, we focused on *ABCB5* as a possible c-MYC target gene. To investigate whether c-MYC directly binds to the *ABCB5* promoter, a ChIP assay was performed with COLO-320 cells. As shown in Figure3B, there were four locations of c-MYC-binding sites (CATGTG) within the *ABCB5* promoter region, spanning from −5000 to +1500 base pairs (bp) with respect to the transcription start site. They were localized at −3415/−3410, −1812/−1807, −87/−82 and +1101/+1106 bp (Fig.3B). We also searched other c-MYC-binding sites (CACGTG and CACGCG), but these binding sites were not present in this region. The results showed that c-MYC bound to two binding sites within the *ABCB5* promoter (−3415/−3410 and −87/−82) (Fig.3C). These results suggest that *ABCB5* is a c-MYC target gene.

### c-MYC inhibitor, 10058-F4, decreased the ABCB5 expression level through the inhibition of c-MYC binding to the *ABCB5* promoter

**Figure 4:**
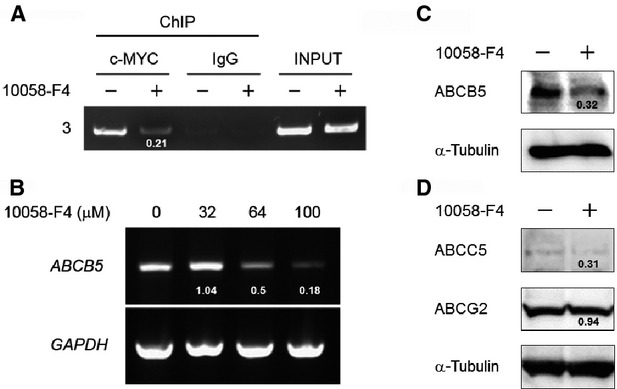
Effect of the c-MYC inhibitor, 10058-F4, on *ABCB5* expression. (A) ChIP analysis was performed on COLO-320 cells treated with DMSO or the c-MYC inhibitor, 10058-F4. The number on the left corresponds to the arrow number in Figure3B. The number under the band in the second lane from the left shows the ratio of ChIP with c-MYC to INPUT band intensity normalized to the value obtained in control cells. c-MYC, mouse monoclonal anti-c-MYC antibody; IgG, normal mouse IgG; INPUT, chromatin DNA of 1/100 quantity used in ChIP. (B) Total RNAs were purified from COLO-320 cells treated with DMSO (vehicle) or 10058-F4 at various concentrations (32, 64 and 100 μM) for 48 hrs, and *ABCB5* and *GAPDH* expression was detected by RT-PCR. The number under each band in the upper panel shows the ratio of *ABCB5* to *GAPDH* band intensity normalized to the value obtained in control cells. (C and D) Whole cell lysates were prepared from COLO-320 cells treated with DMSO (vehicle) or 10058-F4 at a concentration of 64 μM for 48 hrs, and ABCB5, ABCC5, ABCG2 and α-Tubulin expression in whole cell lysates was detected by western blotting. α-Tubulin was used to assess the total amount of whole cell lysates loaded on the gel. The number under the band shows the ratio of ABCB5, ABCC5 or ABCG2 to α-Tubulin band intensity normalized to the value obtained in control cells.

It is well known that c-MYC protein oncogenic activity requires dimerization with MAX 24. To assess the importance of c-MYC-MAX complex-targeted DNA binding on the induction of *ABCB5* expression, we used the c-MYC small molecule inhibitor, 10058-F4, to inhibit the binding of c-MYC to target DNA by preventing c-MYC-MAX heterodimerization 25. A ChIP assay, RT-PCR and western blotting were performed with COLO-320 cells treated with DMSO or 10058-F4 for 48 hrs. 10058-F4 treatment decreased the amount of c-MYC binding to the *ABCB5* promoter (Fig.4A). As expected, 10058-F4 treatment decreased ABCB5 expression at mRNA and protein levels (Fig.4B and C). Furthermore, we investigated the effect of 10058-F4 treatment on the expression levels of other 5-FU resistance-involved ABC transporters, ABCC5 and ABCG2 26. Although 10058-F4 treatment decreased ABCC5 expression, the expression level of ABCC5 protein was very low in COLO-320 cells (Fig.4D, upper panel). ABCG2 protein was expressed in COLO-320 cells, and 10058-F4 treatment did not decrease ABCG2 expression (Fig.4D, middle panel). These results suggest that c-MYC positively regulates the expression of ABCB5 through c-MYC binding to the *ABCB5* promoter.

### ABCB5 is closely involved in 5-FU resistance

**Figure 5:**
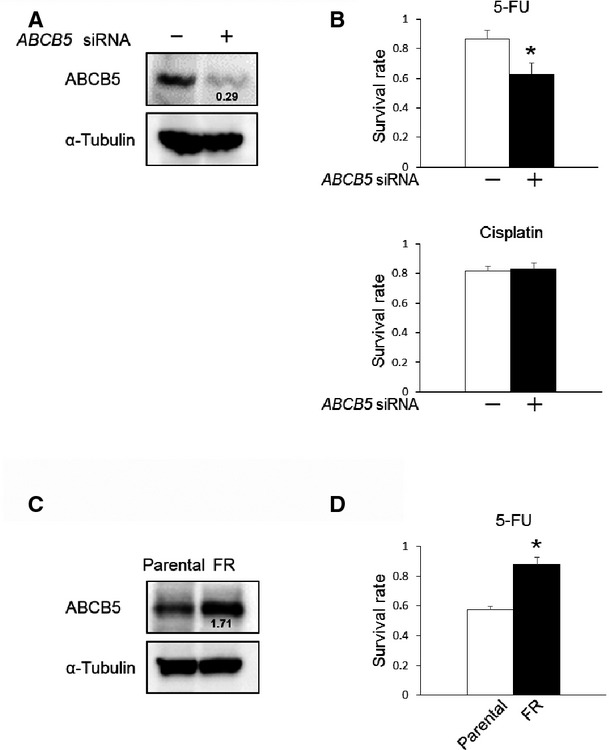
Relationship between ABCB5 expression level and 5-FU resistance. (A) Whole cell lysates were prepared from COLO-320 cells transfected with control siRNA or *ABCB5* siRNA. ABCB5 and α-Tubulin expression in whole cell lysates was detected by western blotting. α-Tubulin was used to assess the total amount of whole cell lysates loaded on the gel. The number under the band in the upper right lane shows the ratio of ABCB5 to α-Tubulin band intensity normalized to the value obtained in control cells. (B) COLO-320 cells transfected with scrambled siRNA or *ABCB5* siRNA were treated with DMSO (vehicle control of 5-FU), 0.9% sodium chloride (vehicle control of cisplatin), 5-FU (5 μM) or cisplatin (5 μM) for 48 hrs, followed by a WST-8 assay to assess the survival rate. The survival rate was normalized to that in the scrambled siRNA or *ABCB5* siRNA transfected cells treated with DMSO or 0.9% sodium chloride as a vehicle control. *P < 0.05, significantly different (*n* = 3). (C) Whole cell lysates were prepared from parental Caco-2 cells or 5-FU-resistant Caco-2 cells, and the ABCB5 and α-Tubulin expression in whole cell lysates was detected by western blotting. α-Tubulin was used to assess the total amount of whole cell lysates loaded on the gel. The number under the band in the upper right lane shows the ratio of ABCB5 to α-Tubulin band intensity normalized to the value obtained in control cells. Parental, parental Caco-2 cells; FR, 5-FU-resistant Caco-2 cells. (D) Parental Caco-2 cells or 5-FU-resistant Caco-2 cells were treated with DMSO or 5-FU at a concentration of 5 μM for 72 hrs, followed by a WST-8 assay to assess the survival rate. The survival rate was normalized to that in parental Caco-2 cells or 5-FU-resistant Caco-2 cells treated with DMSO as a vehicle control. *P < 0.05, significantly different (*n* = 3). Parental, parental Caco-2 cells; FR, 5-FU-resistant Caco-2 cells.

To elucidate the role of ABCB5 in 5-FU resistance, we examined the effect of ABCB5 knockdown on the survival rate following 5-FU treatment in COLO-320 cells. The knockdown of endogenous ABCB5 was confirmed in COLO-320 cells transfected with *ABCB5* siRNA by western blotting (Fig.5A). In ABCB5-silenced COLO-320 cells, the survival rate following 5-FU treatment was significantly decreased compared with that in control cells, and ABCB5 knockdown had no effect on the survival rate following cisplatin treatment (Fig.5B). Moreover, 5-FU-resistant Caco-2 cells presented a high ABCB5 expression level and a significantly high survival rate following 5-FU treatment compared with those of parental Caco-2 cells (Fig.5C and D). These results suggest that ABCB5 is closely involved in 5-FU resistance in human colon cancer cells.

### The combined treatment of 5-FU and 10058-F4 decreased tumorigenicity in nude mice

**Figure 6:**
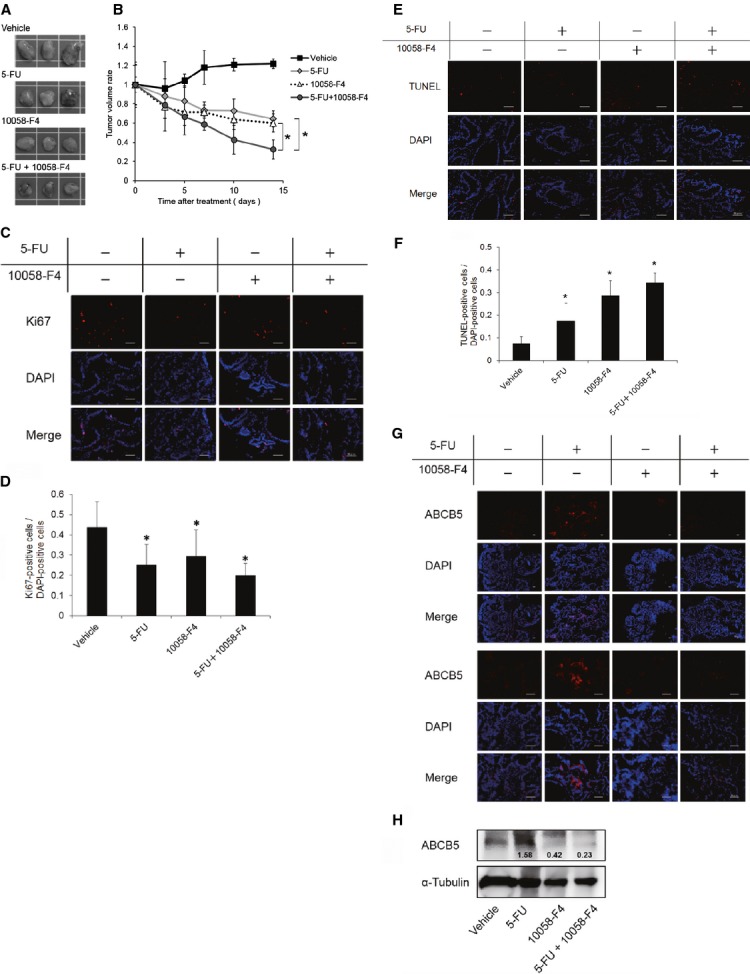
Effect of the combined treatment of 5-FU and 10058-F4 on human colon cancer xenografts. Nude mice were subcutaneously injected with Caco-2 cells. When the tumour volume was about 1000 mm^3^, the mice were treated daily with DMSO as a vehicle control, 5-FU (10 mg/kg), 10058-F4 (20 mg/kg), or 5-FU (10 mg/kg) and 10058-F4 (20 mg/kg) for 2 weeks. Each group consisted of five mice. (A) Representative photographs of tumours following a 14-day treatment with each reagent are shown. (B) Tumour volume was measured with a caliper, and the volume was calculated with the following formula: tumour volume = length × width × height. The tumour volume was measured at day 0, 3, 5, 7, 10 and 14 following the daily treatment with DMSO, 5-FU, 10058-F4, or 5-FU and 10058-F4. *P < 0.05, significantly different (*n* = 5). (C) Representative immunofluorescence staining of Ki67 (red) expression in human colon cancer xenografts following DMSO, 5-FU, 10058-F4, or 5-FU and 10058-F4 treatment for 14 days. Nuclei were stained with DAPI (blue). Magnification ×40; scale bars, 50 μm. (D) Quantitative analysis showed the ratio of Ki67-positive cells to DAPI-positive cells within at least 8 randomly chosen fields. *P < 0.05, significantly different. (E) Representative immunofluorescence staining of TUNEL (red) expression in human colon cancer xenografts following DMSO, 5-FU, 10058-F4, or 5-FU and 10058-F4 treatment for 14 days. Nuclei were stained with DAPI (blue). Magnification ×40; scale bars, 50 μm. (F) Quantitative analysis showed the ratio of TUNEL-positive cells to DAPI-positive cells within at least 8 randomly chosen fields. *P < 0.05, significantly different. (G) Representative immunofluorescence analysis of ABCB5 (red) expression in human colon cancer xenografts following DMSO, 5-FU, 10058-F4, or 5-FU and 10058-F4 treatment for 14 days. Nuclei were stained with DAPI (blue). Magnification ×10 (upper half) and ×40 (lower half); scale bars, 50 μm. (H) Representative western blotting analysis of ABCB5 expression in human colon cancer xenografts following DMSO, 5-FU, 10058-F4, or 5-FU and 10058-F4 treatment for 14 days. The number under each band in the upper panel shows the ratio of ABCB5 to α-Tubulin band intensity normalized to the value obtained in control cells.

To investigate the effect of the combined treatment of 5-FU and 10058-F4 on tumorigenicity in nude mice, a human colon cancer xenograft murine model was developed. As shown in Figure6A and B, 5-FU or 10058-F4 treatment decreased tumour volumes compared with the control. Furthermore, the combined treatment of 5-FU and 10058-F4 significantly decreased tumorigenicity in nude mice compared with 5-FU or 10058-F4 treatment (*P* < 0.05). The ratio of Ki67-positive cells was decreased, and the ratio of TUNEL-positive cells was increased in 5-FU, 10058-F4 and the combined 5-FU and 10058-F4 treatment groups compared with the control (Fig.6C–F). The combined treatment of 5-FU and 10058-F4 tended to decrease the Ki67-positive ratio and to increase the TUNEL-positive ratio the most. Furthermore, 10058-F4 treatment and the combined 5-FU and 10058-F4 treatment decreased the ABCB5 expression level when compared with the control (Fig.6G and H). Conversely, 5-FU treatment alone increased the ABCB5 expression level when compared with the control (Fig.6G and H).

## Discussion

Previous reports indicating that c-MYC was overexpressed in many types of cancers and contributed to drug resistance motivated us to investigate the role of c-MYC expression in 5-FU resistance in this study. First, we performed real-time RT-PCR using surgically resected tumours from 20 patients with colorectal cancer who were subsequently treated with 5-FU-based adjuvant chemotherapy after curative surgery. Our results showed that the *c-MYC* expression level in resected colorectal cancer tissues markedly correlated with recurrence following 5-FU-based adjuvant chemotherapy. Although this study was limited by the small number of cases and short follow-up periods after operation, the results indicate that *c-MYC* expression might be an early recurrence predictive marker for colorectal cancer patients following 5-FU-based adjuvant chemotherapy. Recent studies have indicated that MYCN regulates the transcription of specific *ABC* transporter genes in neuroblastoma 12, and that c-MYC regulates the expression of *ABC* transporter genes in chronic myelogenous leukaemia 13. Given that ABC transporters contribute to the resistance to chemotherapeutic agents by causing the efflux of anti-cancer drugs, we have been suggested that c-MYC increases 5-FU resistance in human colon cancer cells by regulating the expression of ABC transporters. To identify the *ABC* transporter genes regulated by c-MYC in human colon cancer cells, we investigated the effect of c-MYC knockdown on *MDR1*, *MRP1*, *ABCB5*, *ABCC4* and *ABCC5* expression levels, which are closely involved in the resistance to chemotherapeutic agents including 5-FU 17–23. Interestingly, c-MYC knockdown markedly decreased the expression level of *ABCB5*, which promotes doxorubicin transport 20 and is involved in 5-FU resistance 21. Moreover, ChIP assays revealed that c-MYC binds to two binding sites within the *ABCB5* promoter region. c-MYC inhibitor, 10058-F4, decreased the amount of c-MYC binding to its binding site within the *ABCB5* promoter region and led to the decrease in ABCB5 expression at mRNA and protein levels. Furthermore, we examined the change in the expression levels of other 5-FU resistance-involved ABC transporters, ABCC5 and ABCG2 proteins following 10058-F4 treatment. Unexpectedly, COLO-320 cells had very low ABCC5 expression at protein level. Although 10058-F4 treatment decreased ABCC5 expression, it appeared that the decrease in ABCC5 expression had no effect on 5-FU resistance because of its low expression in COLO-320 cells. As expected, ABCC5 knockdown did not decrease survival rate following 5-FU treatment in COLO-320 cells (Fig. S1). ABCG2 was expressed in COLO-320 cells at protein level, and 10058-F4 treatment did not decrease ABCG2 expression. ABCB5-silenced COLO-320 cells showed a decreased survival rate following 5-FU treatment, and 5-FU-resistant Caco-2 cells presented a high ABCB5 expression level and high survival rate following 5-FU treatment compared with those in parental Caco-2 cells. These results suggest that c-MYC confers resistance to 5-FU through regulating ABCB5 expression in human colon cancer cells. Furthermore, *in vivo* study showed that the combined treatment of 10058-F4 and 5-FU significantly decreased tumorigenicity in nude mice compared with 5-FU or 10058-F4 treatment alone. The combined treatment of 5-FU and 10058-F4 tended to decrease the Ki67-positive ratio and to increase the TUNEL-positive ratio the most. *In vitro* study also showed that the combined treatment of 5-FU and 10058-F4 increased the ratio of Annexin V- and PI-positive cells and decreased the expression level of PCNA (Fig. S2A and B). As expected, 10058-F4 treatment decreased the ABCB5 expression level in spite of the presence or the absence of 5-FU treatment. Remarkably, 5-FU treatment alone increased the ABCB5 expression level. This finding is consistent with a recent study demonstrating that the ABCB5 expression level is increased in rectal cancer specimens from patients following 5-FU-based chemotherapy and human colorectal cancer murine xenografts following 5-FU treatment 21. Importantly, we observed that *ABCB5* expression level in primary colorectal cancer tissues did not correlate with the recurrence rate following 5-FU-based adjuvant chemotherapy (Fig. S3). As expected, there was no correlation between *ABCB5* and *c-MYC* expression levels in primary colorectal cancer tissues (Fig. S4). Combined with the results that ABCB5 expression level is increased following 5-FU treatment, we consider that low expression level of *ABCB5* in primary colorectal cancer tissues is because of a lack of 5-FU-based adjuvant chemotherapy, and *ABCB5* expression level may be increased in recurrent colorectal cancer tissues survived following 5-FU-based adjuvant chemotherapy. Although the mechanism by which 5-FU treatment increases ABCB5 expression level remains unknown, we propose two possibilities. One possibility is that some population of colon cancer cells which originally have high ABCB5 expression survive following 5-FU treatment. Another possibility is that 50-FU treatment increases ABCB5 expression in some population of colon cancer cells, and the colon cancer cells with the increased ABCB5 expression can survive. A recent report supports the latter possibility, showing that HIF-1α is up-regulated in tumour cells in response to doxorubicin 27. There is a possibility that 5-FU treatment enhances c-MYC activity and leads to the induction of *ABCB5* expression. Further studies are needed to elucidate the mechanism by which 5-FU treatment increases the ABCB5 expression level.

In this study, we showed that c-MYC confers resistance to 5-FU by regulating ABCB5 expression in human colon cancer cells. Our results suggest that the c-MYC-ABCB5 axis could be a potential therapeutic target in 5-FU-resistant colorectal cancer cells.

## Supporting Information

**Figure S1** Effect of ABCC5 knockdown on the survival rate following 5-FU treatment in COLO-320 cells.

**Figure S2** Effect of the combined treatment of 5-FU and 10058-F4 on Caco-2 cells *in vitro*.

**Figure S3** Relationship between *ABCB5* expression level in surgically resected primary colorectal cancer tissues and recurrence following 5-FU-based adjuvant chemotherapy.

**Figure S4** Relationship between *c-MYC* and *ABCB5* expression levels in surgically resected primary colorectal cancer tissues.

**Table S1** DNA sequence of primers used for real-time RT-PCR.

**Table S2** DNA sequence of primers used for RT-PCR.

**Table S3** DNA sequence of primers used for ChIP assay.
