# Supplementary figures and images for: The c-MYC-ABCB5 axis plays a pivotal role in 5-fluorouracil resistance in human colon cancer cells
Source: J Cell Mol Med. 2015 Feb 17;19(7):1569–81. doi: 10.1111/jcmm.12531 (PMC4511355; doi:10.1111/jcmm.12531)

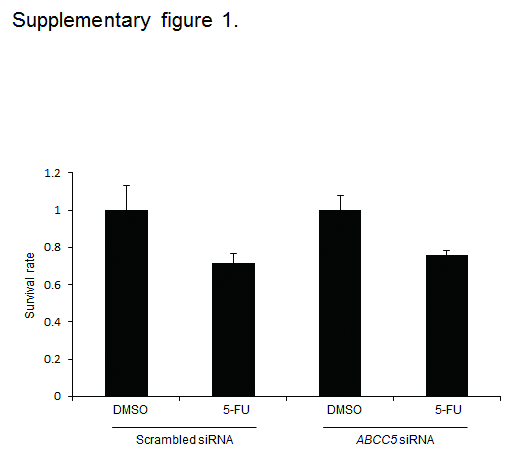

Supplement: Supplementary file 1 [file jcmm0019-1569-sd1.tif]

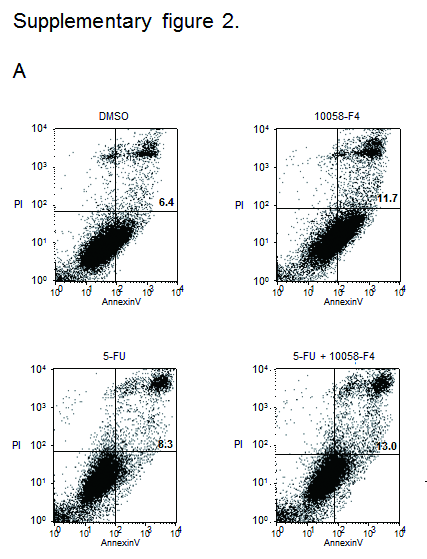

Supplement: Supplementary file 2 [file jcmm0019-1569-sd2.tif]

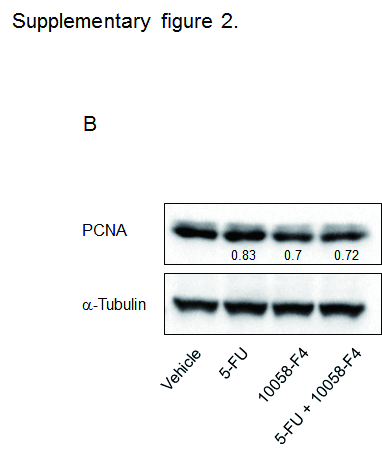

Supplement: Supplementary file 3 [file jcmm0019-1569-sd3.tif]

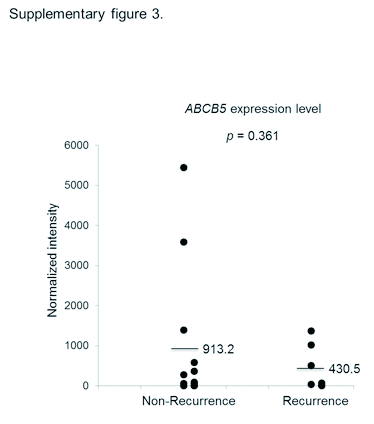

Supplement: Supplementary file 4 [file jcmm0019-1569-sd4.tif]

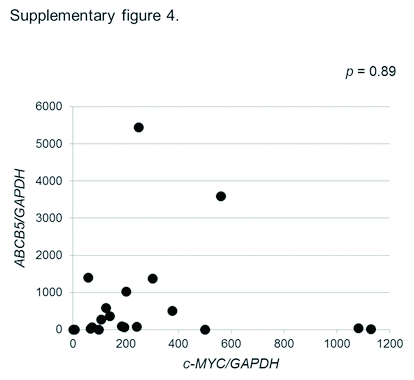

Supplement: Supplementary file 5 [file jcmm0019-1569-sd5.tif]

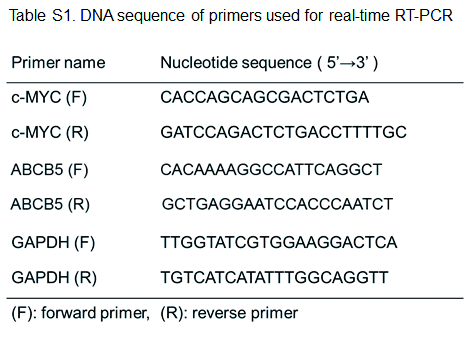

Supplement: Supplementary file 6 [file jcmm0019-1569-sd6.tif]
